# Emotional Intelligence Profiles and Self-Esteem/Self-Concept: An Analysis of Relationships in Gifted Students

**DOI:** 10.3390/ijerph18031006

**Published:** 2021-01-23

**Authors:** Ana María Casino-García, María José Llopis-Bueno, Lucía Inmaculada Llinares-Insa

**Affiliations:** 1 Facultad de Magisterio y Ciencias de la Educación, Universidad Católica de Valencia “San Vicente Mártir”, 46001 Valencia, Spain; ana.casino@ucv.es (A.M.C.-G.); mariajose.llopis@ucv.es (M.J.L.-B.); 2 Facultad de Psicología, Universitat de València, 46010 Valencia, Spain

**Keywords:** gifted, self-esteem, self-concept, emotional intelligence, primary and middle school

## Abstract

The psychological well-being of students affects their academic achievement, social relationships and school coexistence and is something that families worry about. This aspect becomes vital when students have atypical development and/or specific needs. Studies on the impact of giftedness on students’ self-concept and self-esteem offer mixed results. Emotional Intelligence (EI) is a key factor for their well-being that must be developed by educational institutions. This study analyzes the relationships between emotional intelligence profiles and both self-concept and self-esteem of identified gifted students between 8 and 18 years of age who study in regular Spanish schools and non-identified peers. A total of 118 identified gifted and 122 non-identified subjects participated in the study. The Self-Concept Scale Form 5 (AF5), the Rosenberg Self-Esteem Scale (RSES), and the Trait Meta-Mood Scale-24 (TMMS-24) were administered. Clusters of students were identified on the basis of their scores in the three dimensions of EI. Subsequently, the differences in self-esteem and self-concept according to the student’s emotional intelligence profile were analyzed. The results showed a taxonomy of three-cluster profiles in both groups and the existence of differences between profiles of EI in the self-esteem and self-concept dimensions in gifted students, not so in the non-identified group. The results have important implications for education and health professionals, both for the evaluation and for the introduction of adjusted intervention programs in case of vulnerability.

## 1. Introduction

The psychological well-being of gifted students has been an increasing cause of concern in the past 10 to 15 years [1]. There is enough evidence to support that giftedness influences people’s psychological well-being [2], but not always in the same direction; sometimes it protects it (e.g., [3,4,5]), and other times it increases vulnerability (e.g., [6,7,8]). The different results reported throughout the scientific literature may be due to a combination of numerous factors: the personal characteristics of the evaluated subjects, the type of giftedness they present, and the educational fit [1,2,7,9]. An unadjusted level of challenge in gifted children (very low or excessively high) can lead to boredom and wasted potential, as well as diminish well-being and career aspirations [10]. Furthermore, gifted students’ self-esteem and social acceptance can suffer when the educational environment does not meet their needs [9].

In this regard, developing talent in the Spanish educational system continues to be a pending challenge [11], a desire included in the regulations of the Spanish educational law (Ley Orgánica para la Mejora de la Calidad Educativa (LOMCE) [12]. Spain ranks below average in the different evaluations carried out within the Organisation for Economic Co-operation and Development (OECD) countries; it barely has any strong performers or top performers in the Program for International Student Assessment (PISA, levels 5 and 6) [13]. Giftedness remains, to a large extent, unnoticed (0.4% according to Statistics from the Ministry of Education) and still does not receive, for the most part, an adjusted response [14], which can lead to students experiencing learning difficulties, school failure, behavioral problems, etc. [15]. However, not only does guaranteeing the principle of equity in education seem difficult, but also social inclusion becomes complicated. There are generalized myths, stereotypical ideas, and negative social representations in adult society [16], especially among parents of the students [17]. Gifted students sometimes suffer social isolation [15] and are more likely to be involved in harassment situations [18] and cyberbullying than their peers [19], with the victims of these situations suffering from significant levels of psychological affectation [18]. As a group, these children and adolescents also feel greater sadness and less subjective well-being than non-identified students [20]. The lack of psychological adjustment is linked to classroom violence and difficulties in terms of school inclusion [21]. Parents of gifted students also suffer from lower levels of affective well-being [22]. From an inclusive paradigm, the personal, academic, and socio-emotional development of the entire educational community, of all students, including those who are gifted must be ensured [23]. In this regard, recent studies recommend working on the self-concept and self-esteem of gifted students in schools [1,7,17].

Self-concept has been used in different investigations to evaluate the impact of giftedness on the adjustment of gifted children and adolescents, as an indicator of their psychological well-being and adjustment [2]. Self-concept and self-esteem play a fundamental role in people’s lives, their psychological well-being, and their set of social relationships, among other aspects [24]. Although there are different models and theories, all of them underline the importance of the social dimension [25]. We are psychosocial creatures [26], and our self-esteem is based on pleasant social experiences [27]. Through socialization, family and peer groups strongly influence the individual’s lifestyle, set of beliefs, values, etc., that will serve as the basis for the development of these two constructs [24]. To be more specific, the perceptions of gifted children are influenced by the social environment in which they live [25]. Perceived social competence, as well as other domains of self-concept, may be linked to the strategies students use to face or deny their giftedness, in regard to feeling different [28]. For this reason, Emotional Intelligence (EI) becomes a key determining factor, more important than social effectiveness, as it explains the positive effect of social competence on self-esteem [27].

The existing literature has proven that EI is a predictor of important outcomes related to health, education, and relationships [29]. In this regard, EI is positively related to a good psychological adjustment [30]. It is a key factor during adolescence, a period in which the construction of a favorable personal image can have a positive impact on academic performance and social adjustment (e.g., [31,32]). From a contextual and ecological perspective, it seems convenient to carry out studies regarding the different factors in different populations [30], to corroborate and complement these findings [27].

The development of psychosocial skills is essential for the health of gifted students, given their unique social and emotional characteristics and particular experiences (perfectionism, sensory sensitivity, emotional intensity, issues with social interactions, etc.), caused by an asynchronous development [33]. These students live through experiences that differ from the norm, and may struggle with feeling misunderstood by their teachers and classmates, being frequently mis-seen and not seen [34]. The relationship between EI and mental health is complex, and research is needed to deepen its understanding [35]. Skills linked to EI are key for the development of talent, and gifted students often need to find a balance between satisfying their own desires and other people’s perceptions (or the beliefs they have about these perceptions) when doing so [36]. Endless obstacles can prevent the unfolding of their potential: teasing from peers, insane perfectionism, lack of motivation, loss of confidence in their possibilities, invitations from peers not to study, etc. [26]. However, improving EI can build young people’s self-esteem [27]. Analyzing the psychosocial attributes of gifted students to better understand what factors influence it is essential to contribute to their well-being [37]. For this reason, the general objective of this study is to explore different profiles of perceived EI based on its components and to analyze whether there are significant differences in self-esteem and self-concept based on these profiles between gifted and non-identified children and adolescents.

### 1.1. Self-Concept and Gifted Students

Self-concept can be defined as the idea that one has of oneself as a physical, social, and spiritual being. It is the set of abstract self-descriptions that the person creates which, as opposed to self-esteem, are not used as a value judgment [38]. Some authors do not distinguish between these two constructs (e.g., [39]). However, although they are interrelated, research suggests that they are not synonyms [40,41,42]. This component of the personality, although it presents certain stability, is dynamic [38]; that is, it changes over time [2].

Both unidimensional (e.g., [43]) and multidimensional models (e.g., [39]) exist in the conceptualization of self-concept, being the latter the predominant approach at the moment [44]. Different proposals have been developed in which the dimensions are organized hierarchically and divided into other more specific ones [42]. For example, Shavelson, Hubner, and Stanton [39] propose a general self-concept (at the hierarchical, more stable cusp) and distinguish academic from non-academic self-concept. The non-academic self-concept is configured by physical, emotional, and social components that are subdivided into more specific facets. The academic self-concept is subdivided into verbal or mathematical [45], into the perceived competence in school subjects: German, French, mathematics, etc. [46].

Our research follows the self-concept model proposed by García and Musitu [38] which is in line with those developed by previous authors. It is five-dimensional; from a general dimension, self-concept is organized into physical, academic, family, social, and emotional dimensions. The physical dimension describes physical appearance, the individual’s physical condition, sports practice, etc. The academic dimension refers to the perception that the individual has of himself in regard to his quality as a student based on the opinions and qualifications of teachers and his set of skills valued in school contexts. The family dimension focuses on participation and integration within the family; trust and affection, whether the individual is happy, feels helped or supported, whether parents are disappointed by him or criticize him. The social dimension addresses the vision that the individual has of his social performance: their relationship networks, the ease to expand them, desirable qualities related to this dimension, such as being cheerful or friendly. Finally, the emotional dimension contemplates the perception of the emotional state and the specific response to certain situations in which a person of higher rank is involved.

This self-perception of individuals is based on experiences lived with others, as well as on the acknowledgement of their own behavior. Thus, according to Shavelson et al.: “*One’s perceptions of himself are thought to influence the ways in which he acts, and his acts in turn influence the ways in which he perceives himself*” ([39], p. 411). Self-concept is a social construction; self-perceptions are influenced by the social environment [25] and family plays a fundamental role in their formation [38].

Numerous empirical evidence relates self-concept in adolescence to psychosocial adjustment, well-being, and mental health [42]. It also affects other aspects in both gifted and average students: academic performance, successful social relationships, self-confidence, effort, leadership, etc. [37]. The self-concept of gifted students has motivated numerous investigations [2], but the scientific literature offers varied results. Some studies indicate that, in terms of general self-concept, gifted students score higher than (e.g., [44,47,48,49,50], lower than (e.g., [25,51] or equivalent to (e.g., [52,53,54]) their non-gifted peers.

Cognitive abilities have been argued to be highly valued at a social level which explains the above results [25]. Among the factors that have been considered to justify lower scores we find intense sensitivity and high perfectionism exhibited by some of these students [55]. However, Coleman and Fults [25] attribute the differences in results to the theory of social comparison proposed by Festinger [56]: in the absence of general criteria or comparative standards, individuals take others from their environment as a reference to judge their own worth. The specific type of outstanding ability (mathematical, verbal, etc.) has also been pointed out as a factor that may influence this heterogeneity [57].

Regarding the study of the specific dimensions of self-concept, gifted children fundamentally stand out in those areas that involve their intelligence according to Litster and Roberts [44]. The greatest difference, according to the meta-analysis carried out by these authors, is attained in the academic dimension of self-concept. Hoge and Renzulli [48] or Zeidner and Shani-Zinovich [37] found that gifted students had higher academic self-concept scores than their peers. These results are replicated when subdimensions are analyzed, for example, with mathematical academic self-concept [3]. Academic performance and academic self-concept seem to be strongly linked [58,59]. Therefore, a low/high academic self-concept can decrease/increase performance and vice versa. Although students with high potential and low performance may be less likely to follow this trend, they are able to properly describe their academic ability despite their poor results [60], attributing these to the educational system and not to their capacity [61]. Great attention has been given to the Big-Fish-Little-Pond Effect (BFLPE), which indicates that students will have a lower academic self-concept when they are in separate groups with gifted students [62] or with older children, due to skipping grades [63], than when they are in regular classes with lower-ability peers. The authors also attribute this effect to the already mentioned theory of social comparison; a demanding academic environment, with highly capable students, can generate uncertainty in the student’s own ability and induce a decrease in academic self-concept [64]. This decrease is more pronounced during the first weeks of change [65]. In fact, when a student temporarily participates in an enrichment program, this process will become diluted over time when they return to their regular classroom. The student regains confidence and motivation, taking advantage of the experience, which is known as the “splashdown” effect [66].

The support of teachers and peers to promote the high academic level of students is very important for these students to achieve a high academic self-concept and successfully complete their studies. Even twice-exceptional students can score highly if their teachers focus on their particular interests and strengths and provide them with successful experiences [67]. However, low academic self-concept and victimization by peers can frustrate students and negatively affect their academic achievement and success. More specifically, academic self-concept can mediate between the teasing of low academic competence (verbal victimization) and the performance or commitment of gifted adolescents [68].

In terms of the other dimensions, the research results have offered varied results [48]. We summarize the most relevant below. Significant differences have been found in the literature in favor of gifted students in behavioral self-concept (e.g., [48]). However, they frequently score lower in physical self-concept (e.g., [37,44]). They also present less social self-concept (e.g., [37,69]), with exceptions; for example, Lee, Olszewski-Kubilius and Thomson [57] found no differences with respect to the normative sample in how students with high ability perceive their social competence or friendship relationships, although their academic self-concept is better than the social one. These discrepancies between social and academic self-concept have been pointed out by other authors, such as Ross and Parker [70]. Finally, there are no differences in moral self-concept (e.g., [37]). Probably the population of students with high abilities is very heterogeneous, as is the nature of their self-concept and therefore, various profiles can be differentiated [61].

Within the Spanish context, the same heterogeneity of results appears. Pérez and Domínguez [71] found no differences between gifted and normotypical students; gifted students seem to have a good concept of themselves. Nor did De la Torre [72] find differences, although he points out the heterogeneity of the group. Ancillo et al. [73] point out a higher self-concept in identified students, but only the differences in males are significant. Juárez [74] obtained lower results in global self-concept, but higher in academic self-concept. Ortega also recently [75] reported higher scores in academic self-concept for talented students and lower ones in family, but the differences in this last dimension do not reach a degree of significance. This author differentiates profiles according to talents with the intellectual self-concept growing as the complexity of the profile increases, that is, as they stand out in more areas, and points out the large number of factors that can come together in their development. Academic self-concept also correlates with performance and creativity. García, Canuto and Palomares-Ruiz [13] confirm this positive correlation between global self-concept and academic self-concept and performance, and the existence of a relationship between low general self-concept and high intellectual abilities.

In our context, gifted students are not identified through performance. Instead, their potential is assessed through intelligence and creativity tests. Furthermore and as we have previously discussed, the general educational level in Spain is low, as well as the number of excellent students [12,13]. The rates of identified students are also low and specific, adjusted educational responses are scarce. Along with this, the data corresponding to the involvement of gifted students in bullying and cyberbullying situations are higher. In general, adults have incorrect beliefs and negative social representations regarding the gifted group with the well-being of gifted students’ parents being lower. Therefore, we propose the first hypothesis.

**Hypothesis** **1.** *Identified children will have higher scores in academic self-concept, similar scores in emotional self-concept, and lower scores in physical, family, and social self-concept compared to non-identified students.*

### 1.2. Self-Esteem and Gifted Students

Self-esteem is “*a favorable or unfavorable attitude toward the self*” ([76], p. 15). It is “*the evaluative component of the self-concept*” ([77], p. 298). This affective dimension could be considered the final conclusion of a self-evaluation process: I like myself, I am satisfied with myself, I am valuable; or I don’t like myself, I feel unsatisfied, I’m not worth it [38]. It is closely related to how our significant “others” treat us and to the triumphs or successes achieved in life [46]; for example, it seems to be the result, in part, of good school achievement [78].

This construct has been assessed in different ways and with different instruments, usually self-reports [77]. Rosenberg [76] was one of the first authors to propose that it be evaluated using a holistic approach [79], and not based on specific abilities or qualities as proposed by other models (e.g., [43]). The Rosenberg Self-Esteem Scale (RSE) scale is one of the most used instruments to measure the general attitude that a person has toward their importance or worth [80], their general feelings about themselves, and it is considered one of the best measures of global self-esteem [77].

Global self-esteem is a highly relevant personal cognitive variable for psychological well-being [81]. According to these authors, the level of competence of an individual in a specific area (for example, academics) will not affect the global self-esteem of the individual, unless this facet is important for them. However, self-esteem is a catalyst in the developmental process of talent [82,83].

During childhood, self-esteem is positively related to academic performance [84], negatively related to depression [85], and predicts anxiety [84]. During the elementary school ages, it seems that there is a bidirectional relationship between self-esteem and subjective well-being at school [86]. However, it is in adolescence when it truly becomes key, given that the development of identity is highly influenced by relationships with others. According to the sociometer model of self-esteem [87], the self-esteem system observes and evaluates the reactions of others and warns the individual about the possibility of being excluded, helping them maintain their connection with other people. The social basis of self-esteem is social competence [27]; negative social experiences degrade self-esteem [88]. Adolescents with high self-esteem are cooperative, work better in teams, experience jealousy-loneliness to a lesser degree, show high tolerance to stress, high self-demand and perseverance, high intelligence, and social inclusion [41]. Self-esteem plays an important role in parent-child interaction and in improving the subjective well-being of the adolescent [89].

Studies that have been conducted with gifted students offer varied results. Gifted students generally score lower (e.g., [90] in young children, 6–8-years-old; [91]) or higher (e.g., [49,50,92,93]) than their non-gifted peers. Other studies have found no differences between the self-esteem of identified and non-identified students (e.g., [94,95,96]). General self-esteem positively correlates with motivation and academic achievement in gifted students [97]. In addition to social comparison, other factors can influence the results; for example, the system used to designate students as highly capable. Kroesbergen et al. [10] point out higher levels of self-esteem in those high-ability students nominated by their teachers; the same happens with those who present higher performance. On the other hand, having low self-esteem is one of the frequent reasons for visiting the school counselor among this group [7].

Within the Spanish context we find few studies. Some of them indicate that gifted adolescents obtain high levels of self-esteem [73,98]. Another study found that in 9- to 12-year-old children, self-esteem correlates with academic achievement, with identified girls obtain lower scores than their male counterparts [15]. García Agius [99] and Ortega [75] did not find differences when comparing 6- to 12-year-old students with and without high ability. However, and despite the fact that the differences increase with age [15], we have not found studies with Spanish adolescents.

Given that there seems to be a two-way relationship between self-esteem and subjective well-being at school and that negative social experiences diminish the individual’s appreciation of oneself, especially in adolescence, and considering the figures for bullying among gifted students in our country, the greater sadness of this group and their lower levels of affective well-being compared to their peers in the studies carried out in our community, we formulate our second hypothesis as follows.

**Hypothesis** **2.** *Identified gifted students will have lower self-esteem scores than their non-identified peers.*

### 1.3. Emotional Intelligence and Gifted Students. Relationship between Emotional Intelligence, Self-Concept, and Self-Esteem

EI was defined by Salovey and Mayer in 1990 as “*the recognition and use of one’s own and others’ emotional states to solve problems and regulate behavior*” ([100], p. 189), and is considered a catalyst in the process of transforming potential into talent [82].

There are different conceptualizations of EI. The cognitive ability model considers that there is a system of different mental skills that process emotional information. On the other hand, the approach based on EI being a non-cognitive trait defends that it is a disposition that occupies the lower levels of the personality hierarchy [101].

The Mayer and Salovey [102] model is one of the most influential and rigorous. It proposes four branches: the individual perceives emotions with precision; uses them to facilitate thinking; understands emotions and their meanings; and manages emotions within and with others. This approach is located in the first model [103].

Performance tests are often used to assess the real level of EI as a cognitive ability, with self-reports being commonly used to measure trait EI [101]. One of the most used questionnaires is the Trait Meta-Mood Scale (TMMS [104]). This self-report of the reflective processes that accompany moods is based on the model of Salovey and Mayer [100], and provides an index of the beliefs that the subject has about their emotional abilities. It evaluates three factors: attention (the attention paid to one’s moods); clarity (the understanding of one’s moods), and repair (the regulation of moods) [105]. When assessed with self-reports, EI appears to be more strongly associated with mental health [106].

In children and adolescents, EI is positively related to psychological adjustment [30], subjective well-being [107], intrinsic motivation [108], and academic achievement [109,110]. It is also negatively correlated with social anxiety [107], cybervictimization, and suicide risk [111]. It can even dampen the negative impact of very intense emotions on psychological well-being, such as the fear of terrorism in adolescents living in high-risk areas [112].

A better self-concept is associated with a high EI profile [113]. However, each of the EI dimensions carry a different weight [114]. In the study carried out by Martínez-Monteagudo et al. [114] with Chilean adolescents, four profiles were identified: high EI, low EI, high attention and low repair, and low attention and high repair. The highest scores in self-concept were obtained by adolescents who had high scores in all three dimensions and those in the low attention and high repair emotional profile. Landa, et al. [115] found positive correlations in nursing students between all the self-concept scales and the clarity and repair emotional factors. In a study of university students with reduced mobility, Suriá-Martínez Ortigosa and Riquelme [113], identified three EI profiles in a cluster analysis: a group of young people with high overall EI scores, another with high clarity and repair scores, and a last group with low scores in all three dimensions. Students with motor disabilities obtained lower scores in self-concept than their peers. The group with a high EI profile obtained the best results. There were hardly any differences between scoring high in the three dimensions or only in clarity or repair for family, social, or emotional self-concept; the attention dimension did not seem relevant. However, in academic self-concept it was vital that all three dimensions have a high score.

EI is a determinant of self-esteem and it may therefore be essential to provide social experiences that favor it [27]. In general, EI predicts self-esteem in adolescents [116]. According to the study carried out by these authors, when analyzing the dimensions separately, clarity and repair also seem to be predictive. In another study [117], only clarity predicted self-esteem, while repair affected satisfaction with interpersonal relationships; both dimensions were found to influence positive emotions. It seems that adolescents with high scores in these two dimensions tend to feel more positive affect during the self-assessment process and to assess themselves better, which in turn provides them with greater satisfaction with life; that is, the average self-esteem in this process [118]. In kindergarten children, social-emotional learning preventive programs improve self-esteem [119].

There is no relationship between EI (trait) and cognitive ability [120]. However, gifted students can experience emotional overexcitability [121], great emotional intensity, strong empathy, and great affective expression [33]. In recent meta-analyses [122,123], the different results of studies on EI in gifted students are verified. In general, these students present high EI scores and slight differences, in their favor, compared to normotypical students. However, they may excel in some aspects of EI and have lower scores in others. For example, non-gifted students are more effective at stress management [122]. Gifted students obtain higher scores in moods, but lower in intrapersonal skills; it is difficult for them to describe and share their feelings [124]. In general, the scores obtained by these students in the ability tests are higher than with self-reports [125].

In regard to samples from the same Spanish region, gifted students presented a total EI, perception and management of emotions significantly higher than their normotypical peers when taking ability tests [126]. However, the self-perceived EI was significantly lower [20]. In both studies, clarity obtained a significantly lower score.

Therefore, we propose the following hypotheses:

**Hypothesis** **3.** *There will be different EI profiles depending on the attention, clarity, and repair dimensions. These profiles will vary depending on whether students are identified as being gifted or not.*

**Hypothesis** **4.** *There will be significant differences in the dimensions of self-concept and self-esteem in both groups depending on the EI profile and they will be more pronounced in gifted students.*

## 2. Materials and Methods

### 2.1. Procedure and Participants

This was a cross-sectional study with a sample composed of 240 Spanish students between the ages of 8 and 18 (M = 10.56; SD = 2.39). Convenience sampling was used and all participants in this study were conveniently available to participate in the study and did so voluntarily and. The criteria used for the gifted sample was to have a signed report from a licensed psychologist. The procedure was as follows: First, the purpose and the study were explained to both parents and their children. Second, parental consent and student approval (when the student was at least twelve years old) was obtained and signed before the student began completing the questionnaires. Three scales were completed by the two samples and measures of sociodemographic information were collected. The participation in the study was anonymous and voluntary and students could abandon the study whenever they wanted. The administration of the questionnaires was carried out by two investigators and the researchers checked that all questionnaires were fully completed.

Of the total sample, 30% were female. The different educational levels of the participants were primary education (69.2%), secondary compulsory education (27.1%), post-compulsory education (2.9%), and university (0.8%). The total sample was divided into two samples: gifted students (sample 1 = 49.2%) and unidentified students (sample 2 = 50.8%).

Sample 1 consisted of 118 gifted students (84 male; MAge = 10.64, SDAge = 2.42) with the following educational levels: primary education (68.6%), secondary compulsory education (28.8%), post-compulsory education (1.7%), and college education (0.8%). A total of 95.8% of the students attended extracurricular activities. The majority (40.7%) participated in two extracurricular activities, 32.2% participated in one single activity, and 24.6% participated in three-five activities. Moreover, 11% gifted students had skipped grades.

Sample 2 consisted of 122 unidentified students (66.4% male; MAge = 10.53, SDAge = 2.37) with the following educational levels: primary education (68.8%), secondary compulsory education (26.2%), post-compulsory education (4.3%), and college education (0.7%). A total of 96.7% of the students attended extracurricular activities. The majority (50%) participated in only one extracurricular activity, 36.9% participated in two activities, and 9.8% participated in three or four activities.

### 2.2. Measures

#### 2.2.1. Emotional Intelligence

Trait Meta-Mood Scale-24 (TMMS-24) by Fernández-Berrocal, Extremera, and Ramos [127] was administered to analyze emotional intelligence. This was a shortened adapted version of the scale from Salovey et al. [104]. The psychometric proprieties were analyzed by Salguero et al. [128]. TMMS-24 has 24 items and assesses the students’ perception of their abilities regarding the attention they pay to their own emotions, how they discriminate between them and how they perceive their ability to regulate them. This scale consists of three subscales with eight items each: attention (αSample 1 = 0.82; αSample 2 = 0.78), clarity (αSample 1 = 0.86; αSample 2 = 0.83), and repair (αSample 1 = 0.77; αSample 2 = 0.72). Attention is the ability to perceive one’s own emotions and those of others (e.g., “I pay close attention to how I feel”). Clarity assesses the ability to understand emotional information (how emotions combine and progress over time) and to understand emotional meanings (e.g., “I am usually very clear about my feelings”). Repair refers to the skill to change feelings and those of others as well as to promote understanding and personal growth (e.g., “Although I am sometimes sad, I have a mostly optimistic outlook”).

#### 2.2.2. Self-Esteem

We evaluated student self-esteem with the Global Self-esteem Scale of Rosenberg [76]. We used the Spanish version of Atienza, Moreno, and Balaguer [79]. This scale evaluates global self-esteem and asks about general feelings regarding the self. It has a single factor with ten items and a four-point Likert response scale (1 = strongly disagree, 4 = strongly agree). Five of those items are positive feelings (e.g., “On the whole, I am satisfied with myself”) and the other five items are negative feelings about the self (e.g., “I feel I do not have much to be proud of”). Cronbach’s alpha values were 0.83 for the gifted sample and 0.82 for the unidentified sample.

#### 2.2.3. Self-Concept

To measure student self-concept, we used a brief version of the AF5 Scale [38] of García-Grau et al. [129]. It is a 20-items measure which evaluates the “cognitive and social construction that is developed throughout life and is shaped by the set of characteristics that are consciously assumed by the individual” (p. 151). The scale has five dimensions: academic self-concept (e.g., “I am a good student”), social self-concept (e.g., “I am a friendly person”), family self-concept (e.g., “I feel happy at home”), emotional self-concept (e.g., “A lot of things make me nervous”), and physical self-concept (e.g., “I like my physical appearance”). It uses a five-point scale ranging from 1(strongly disagree) to 5 (strongly agree). The scale’s reliability was satisfactory in two samples (Sample 1 αAcademic = 0.85, αSocial = 0.83, αEmotional = 0.71, αFamily = 0.81, and αPhisical = 0.61; Sample 2 αAcademic = 0.83, αSocial = 0.70, αEmotional = 0.72, αFamily = 0.73, and αPhisical = 0.64).

### 2.3. Data Analysis

Data analysis was performed using SPSS software version 26 (SPSS, Chicago, IL, USA).

First, Confirmatory Factor Analysis (CFA) was carried out to three scales. After the dimensionality of the questionnaire had been clarified the mean and standard deviation of the factors were calculated and used to provide insights between the different groups (descriptive analysis). Moreover, the Student’s *T*-test was used to compare gifted and unidentified students.

Second, we calculated correlations as a previous step to the creation of profiles. A two-step Cluster Analysis was executed to analyze profiles of students of both groups (gifted and unidentified students). This method is an exploration tool designed to discover the natural groupings of a data set [130]. Euclidean distance as the proximity measure was used to identify the cluster solution.

Third, to analyze statistically significant differences in terms of self-concept and self-esteem between EI profiles, we used the variance ANOVA analysis. Then, we used Tukey’s Post Hoc Test to identify where the differences were present.

## 3. Results

### 3.1. Descriptive and Preliminary Analysis

First, CFA was carried out to examine the dimensionality of the three scales. This model presents in the three scales an adequate fit to the data (Table 1). We analyzed students’ characteristics concerning emotional intelligence, self-esteem, and self-concept. In Table 2 we present the mean and typical deviation of the total sample, both gifted students and unidentified students. In the total sample, the mean score of the dimensions of the EI were medium for attention (minimum = 1; maximum = 5) and high for clarity (minimum = 1.25; maximum = 5) and repair (minimum = 1.50; maximum = 5). Self-esteem was high (minimum = 2.20; maximum = 5) and self-concept was medium-high in emotional self-concept (minimum = 1; maximum = 5) and high in the other dimensions (minimum_Academic_ = 1.50; maximum_Academic_ = 5; minimum_Social_ = 1; maximum_Social_ = 5; minimum_Family_ = 1; maximum_Family_ = 5). We compared the mean scores between the two groups of students (gifted and unidentified) (Table 2) and we compared the scores of both groups (gifted and unidentified). Table 2 shows that there were statistically significant differences in terms of emotional intelligence clarity and self-esteem. The data also evidences that there were significant differences in the social, family, and physical self-concept. Unidentified students had higher scores than gifted students on the overall dimensions. These results supported H2 and partially supported H1.

### 3.2. Emotional Intelligence Profile Identification

To test Hypothesis 3, Cluster Analyses for gifted and unidentified students were carried out to identify emotional profiles. Correlations between all dimensions of EI were analyzed for the examination of multicollinearity (Table 3). Correlations were small or moderate (0.47 or less), the dimensions were conceptualized as different in the framework, and three factors of EI were included in the cluster analyses [131].

**Figure 1:**
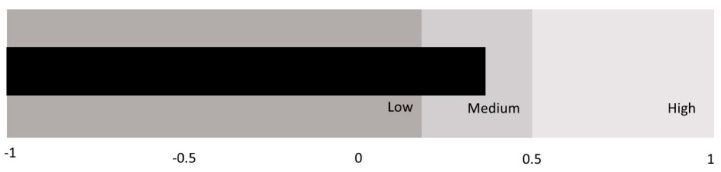
Goodness of the gifted students’ model of the two-step cluster.

**Figure 2:**
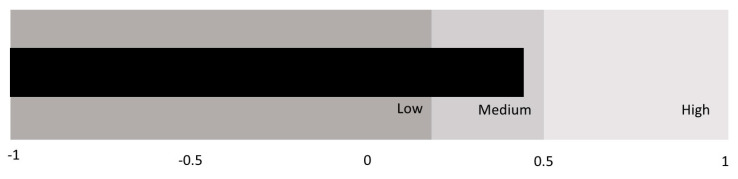
Goodness of the unidentified students’ model of the two-step cluster.

To find similar patterns of emotional intelligence, we conducted a Two-Step Cluster analysis. The Cluster resulted in three groups based on the weight of each EI dimension (attention, clarity, and repair) in both groups of students (gifted and unidentified) with adequate cohesion (Figure 1 and Figure 2). The profiles were different in gifted and unidentified students (Table 4). In gifted students, the first group (Cluster 1) consisted predominantly of high repair, with medium-high clarity, and medium attention. A total of 89.8% of the gifted students were in this group. The second group (Cluster 2) held 8.5% of gifted students who had high clarity and medium-low repair and attention. The third group (Cluster 3) was characterized by high attention and medium clarity and repair. Only 1.7% of gifted students were in this group. These results supported H3.

**Figure 3:**
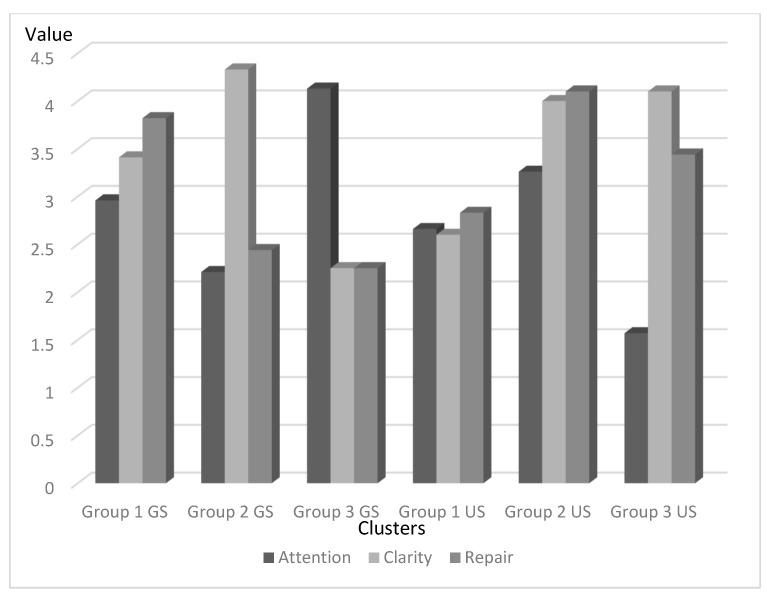
Representation of the three-cluster EI model for gifted and unidentified students. GS = gifted students; US = unidentified students.

In regard to unidentified students (Figure 3), the first group (Cluster 1) mostly scored medium-low on all of the EI dimensions. This group included 19.6% of the unidentified students. The second group (Cluster 2) contained 73% of unidentified students who had high clarity and repair with medium attention. The third group (Cluster 3) was characterized by low attention with high clarity and medium repair. Only 7.4% of unidentified students were in this group.

### 3.3. Differences between EI Profiles of Gifted and Unidentified Students with Self-Esteem and Self-Concept Dimensions

**Figure 4:**
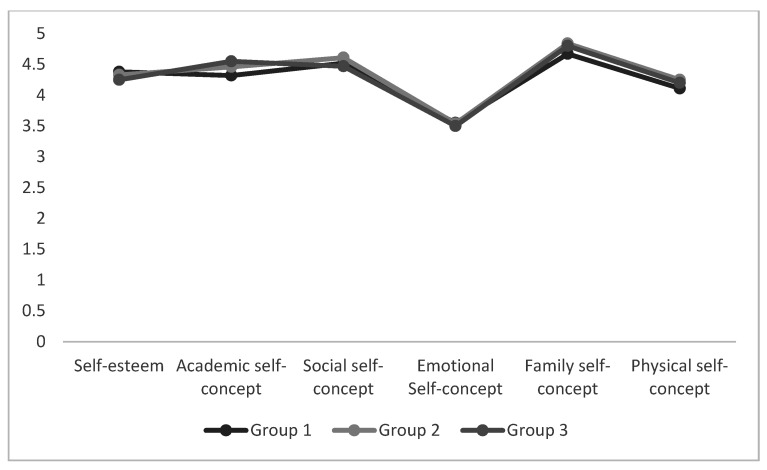
Mean Scores of Self-Esteem and Self-Concept as a Function of Unidentified Profiles of Emotional Intelligence (EI).

**Figure 5:**
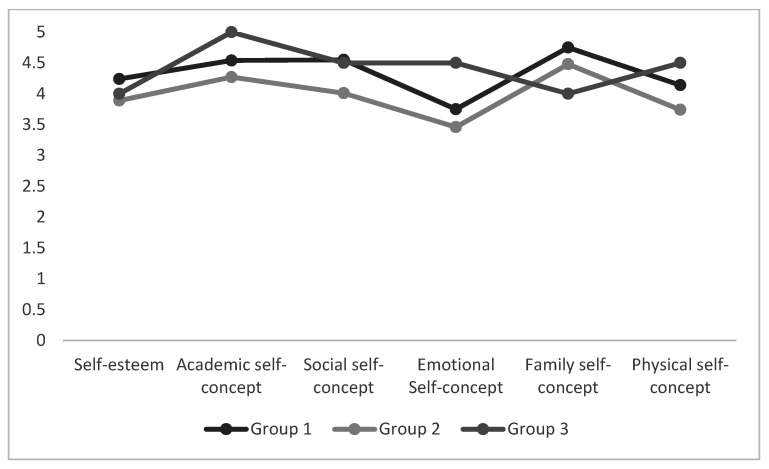
Mean Scores of Self-Esteem and Self-Concept as a Function of Gifted Profiles of Emotional Intelligence (EI).

To test whether the emotional profile was significantly different in self-esteem and self-concept, we conducted an analysis of variance ANOVA with Tukey post-hoc analyses. Each student was assigned to a cluster and then we compared their self-esteem and self-concept scores. Results revealed that there were no significant differences between the three emotional intelligence groups with either self-esteem or self-concept in unidentified students (Figure 4). However, we observed significant differences in the three profiles of gifted students in self-esteem (F_(2115)_ = 2.95; *p* = 0.05), social self-concept (F_(2115)_ = 3.90; *p* = 0.02), and physical self-concept (F_(2115)_ = 3.50; *p* = 0.03). These results indicated that there were statistical differences in the levels of self-esteem and some dimensions of self-concept among the different emotional profiles. Figure 5 shows the differences between emotional intelligence profiles in regard to self-esteem and self-concept. Students in group 1 had higher scores in self-esteem (M_G1_ = 4.24; and M_G2_ = 3.89; M_G3_ = 4), and social (M_G1_ = 4.55; M_G3_ = 4.5; and M_G2_ = 4) and physical self-concept (M_G3_ = 4.50; M_G1_ = 4.14; M_G2_ = 3.74). The findings partially confirm H4. We did not find differences in self-esteem and self-concept according to the emotional intelligence profile in unidentified children.

## 4. Discussion

In this work we analyzed self-esteem and self-concept in a sample of both identified and non-identified gifted Valencian children and adolescents based on their EI profile. The physical, academic, emotional, family, and social dimensions were explored for self-concept. Self-esteem was assessed with a global measure of the value attributed by the individual. To understand the subject’s perceived emotional intelligence, a self-report that evaluated three dimensions was used. These dimensions corresponded to the attention paid to the emotions felt, the understanding of them, and the regulation strategies used to manage these feelings.

Regarding the first of the constructs evaluated, self-concept, relevant differences can be observed between the two groups in terms of family, social, and physical self-concept, with identified gifted students obtaining lower scores. Contrary to expectations, there are no differences in academic self-concept. Thus, H1 is partially accepted.

Focusing on academic self-concept, gifted students achieve good scores, but these do not differ significantly from those obtained by their non-identified peers. Our results are different from those obtained by Zeidner and Shani-Zinovich [37] and Ortega [75]. However, there are several explanations that can account for this. Israeli students from both regular heterogeneous schools and special homogeneous schools all participated in Ministry programs for gifted students. Children stand out academically if the system adapts its education to their strengths and needs and allows the development of talent, even for twice-exceptional students [67]. The same can be said of Ortega’s [75] research. The participants in this Spanish study attended a school that had a protocol for detection and offered attention to the specific educational needs of students with high intellectual abilities, with all of them receiving support, which does not usually happen in our country [11]. In addition, the development of talent requires students to be willing to persist in spite of facing challenges and to take advantage of growth opportunities [26]. Participants in our study were identified using tests that assessed ability and potential, not performance. Therefore, among the evaluated students there were some who got high or average grades, and some who were even failing.

The development of academic self-concept begins very early, during Grades 1 and 2 of elementary school, even before children start receiving proper grades [132]. Although studies link academic self-concept with performance [133], feedback from teachers and the information provided by their parents and loved ones also influences its development [132]. Children need support to achieve excellence [134,135]. In fact, Kroesbergen et al. [10] obtain results similar to ours in regard to gifted students. However, in the “students nominated by their teachers” and “high achievers” subgroups, children were found to display better academic achievement and school enjoyment. When teachers recognize the potential and talent of their students, their educational well-being and adjustment is better.

Furthermore, because our students did not participate in gifted programs, they were not able to experience the “splashdown effect” upon returning to their regular classroom. They did however belong to a family association whose goals were to raise awareness among the educational community, and society in general, of the particular educational needs that their children may present and that the educational system does not always meet. They participated in activities, but they have no connection with the school context, there is no connection with their teachers.

In regard to family self-concept, support and strong protection from family members is especially important in these students’ lives. However, parents face unusual challenges during their upbringing [136]. Our results go in the same direction as those obtained by Ortega [75]. In his work, family self-concept scores were also lower, but not significant. Furthermore, families had the school’s support. However, most of the parents participating in our research belong to associations where they coincide with other parents who fight for a better education for their children and collaborate in informational, advocacy, and training actions. They also receive counseling. It therefore seems unlikely that these parents would exert undue pressure on the students. Nevertheless, they report lower subjective well-being than other parents of non-identified children [22]. Adults, and more specifically the classmates’ parents, have negative social representations regarding giftedness [17]. Thus, gifted children may feel somewhat different from their peers, ashamed of not being able to resolve their difficulties on their own, and “guilty” of their parents’ concerns. The most beneficial support is sometimes that which is invisible to the recipient [137]. Lack of autonomy can lead to loss of confidence in the individual [26].

In terms of social self-concept, our results are in line with those obtained by Kroesbergen et al. [10] with younger children, or with those collected by Litster and Roberts [44] in their meta-analysis. In the literature, the differences are attributed to the fact that these students tend to have different interests from those of their peers, making it difficult for them to share hobbies, games, and conversational topics. However, in the study by Ortega [75], this difference does not appear. Being talented in the school where the research was carried out did not make children feel different, given the high number of identified students due to the use of a detection protocol. Furthermore, it ensured participation in certain educational activities. Individuals have a fundamental psychological need to belong to a group and be accepted by their peers, to establish and maintain lasting interpersonal connections [138]. This need becomes more acute during adolescence, a stage of personal growth where one of the great challenges is to acquire the security that one can make friends for life [82]. Effective schools convey the importance of academic achievement to their students. A peer group that values performance will encourage student engagement, but an environment of marked anti-intellectualism will invite students to excel in other types of activities considered more valuable than studying. Popularity dictates the values of the group [36].

According to the review carried out by Neihart [2], children who study full time in a differentiated education have a lower self-concept than those who study part-time. In our study however, students attended regular classes full-time and followed the prescribed curriculum, often without any adaptations [14,17]. From an inclusive educational paradigm, having different interests should not be a barrier to participation or a reason for exclusion [139]. Schools are microcosms of society, where students learn social and behavioral norms [36], therefore they must offer safe environments; the middle school years are difficult, and students need teachers that are sensitive to their needs [26]. Teenage children go through an identity crisis in which they are tremendously concerned about the perceptions of others and their beliefs, aware of possible ridicule; some adolescents may be cruel and intolerant of different peers and exclude them from the group [140]. Gifted students may reject their identity in order to not stand out and be accepted by their peers. The negative reflection of themselves that they perceive in others can lead them to hide [34]. Social identities are derived from personal ones; if the reference group does not value academic activity, the motivation to excel in class assignments can diminish or the public image of the assertive adolescent who strives for learning may suffer [36].

The quality of the social relationships that a person has is one of the strongest predictors of their well-being [141]. In children and adolescents, peer relationships are frequently used as an indicator of their adjustment [2]. Especially linked to self-concept, the importance of friendships increases with age [142]. Social self-concept also correlates positively with academic achievement or the approval of teachers and classmates, and negatively with disruptive behaviors, aggressiveness, and depressive symptoms [38]. In some studies [20], gifted students have shown to have lower levels of emotional well-being than their non-identified peers and to feel more sadness and loneliness [143]. They also suffer negative discrimination when they are excluded by their classmates [144], are involved more often in bullying situations and, a significant percentage (25%) considers that teachers have, in a certain way, encouraged them to be victims of this [18]. Paradoxically, one of the most important aspects of well-being in childhood and adolescence seems to be the support of teachers and school staff [142], which also positively influences conflicts between peers [145]. The effective resolution of conflicts with peers and friends is associated with adolescents’ happiness and satisfaction, which is decreased by negative emotions [145].

The scarce training of teachers regarding gifted students [17] allows the survival of myths and stereotyped images. The small number of identified students leads us to think that only those who stand out significantly from the rest are evaluated. Students who verbalize that they feel “different” also seem to have a more negative image of their social adjustment [49]. However, the lack of teacher training and the existence of negative stereotypes seem to be widespread [146]. For example, Australian teachers in training associated giftedness with higher intellectual capacity and less adjustment [147]. Their German and Austrian counterparts in training also linked higher abilities to higher intellect and greater social maladjustment, and they showed lower self-efficacy to teach this type of students and a lower motivation [148].

A negative social self-concept can lead to mental health problems [149]. The importance of the family and social dimensions is indisputable. The enhancement of social networks on Internet through multiple electronic devices and various applications has magnified its significance (e.g., [150]).

Regarding the second hypothesis, gifted students have significantly lower scores in self-esteem than their non-identified peers, which confirms our initial expectations. Thus, H2 is accepted. Our results coincide with those obtained by Kroesbergen, et al. [10] with even younger children, 6- to 8-year-olds. According to these authors, when children grow up, if the educational environment does not meet their needs, their self-esteem suffers. At certain times in life, self-assessments move from the center to the periphery (family self-esteem, social self-esteem, etc.), depending on the importance given by the subject to the reference groups and their motivation toward what their prioritized social role is supposed to be, even going as far as to undermine their individual identity [149]. This will have clear practical implications to guide interventions. In adolescence, the student is torn between the adults’ appraisal and the acceptance of peers, and being intelligent or academically good is not highly valued or popular at these ages [36]. Feeling “different” produces a decrease in self-esteem and a greater number of difficulties in relation to peers [49]. Bullying/cyberbullying also has a negative effect on the self-esteem of victims (e.g., [151]). Self-esteem and cyberbullying maintain strong links according to the role, the context, etc. [152]. People with low self-esteem seem more permeable to situational influences and blind feedback [153]. Again, networks magnify these effects [154].

The third hypothesis focused on the existence of different profiles, combinations of the dimensions of the chosen EI model (attention, clarity, and repair) depending on the weight of each of them, different for identified and non-identified students. The cluster identified three different profiles in each group, so H3 is accepted. On the one hand, two fairly similar profiles are defined in both groups, differentiated by the scores in clarity (lower in gifted students): medium attention, high clarity in non-identified and medium-high for gifted, and high repair. These profiles are presented by the highest percentage of students in both groups. On the other hand, we found four very different profiles, two in each group. Most striking are the low levels of repair of the two minority profiles in gifted students. Profile 2 of gifted students presents medium-low attention, high clarity, and low repair. In profile 3, low repair is accompanied by high attention and low clarity. The gifted group’s scores on clarity are significantly lower overall than the non-identified group. In the non-identified group, profile 1 presents medium-low attention, medium-low clarity, and medium-low repair. Meanwhile, profile 3 presents low attention, high clarity, and medium-high repair. In principle, in the profile with the highest EI there would be a higher percentage of gifted students, but the low scores in repair in the other two profiles would place these individuals in a situation of vulnerability, which is consistent with the conclusions of the meta-analysis of Ogurlu [123]. Gifted students, as a group, present better results in EI than those not evaluated, but when the components are analyzed separately, a small percentage of misaligned profiles appear. It would also be necessary to increase the clarity dimension.

Finally, there are no differences between profiles in self-concept or self-esteem in the group of non-identified students, but there are differences between the profiles of the group of gifted students: in self-esteem, social self-concept, and physical self-concept between profiles 1 and 2, in favor of the first. Thus, H4 is partially accepted. The results are consistent with the previous literature. Clarity predicts self-esteem [116,117] and repair predicts self-esteem [116] and satisfaction with personal relationships [117]. They are also in line with the results of other studies carried out in other educational and collective stages (e.g., university students with and without motor disabilities [113]).

Our results show better scores in some dimensions of self-concept and self-esteem as a function of the emotional profile. High EI corresponds to profile 1 of our study. Thus, as a group, gifted students would achieve a good adjustment given that a higher percentage of the non-identified are included in this group. However, it would be convenient to intervene with programs to improve emotional intelligence in vulnerable students (profiles 2 and 3), especially aimed at improving the regulation of students, and analyze other factors, such as perceived social support and their educational inclusion. “Thus, when emotional problems occur, one needs to look for inconsistency between the social and emotional needs of gifted individuals, and their social and educational environment, rather than assuming emotional deficiencies in the gifted population” [123] (p. 9).

Our work offers several contributions to this growing body of research. First, there are hardly any studies comparing self-concept and self-esteem of identified students who study full time in regular classrooms with their non-identified counterparts. Most studies focus on the self-concept of the gifted group, or compare it to high achievers or participants in enrichment programs or special education [37]. Additionally, the type of school these students attend, and the social comparison group can affect results [44]. Second, most studies focus on children [37], even though adolescence is a key stage in the construction of one’s identity [140]. Our study includes both identified and non-identified children and adolescents who study full time in regular classrooms. In line with the latest trends, it also includes comparative measures of the different dimensions of self-concept and its affective assessment, global self-esteem. Third, studies carried out on self-concept and self-esteem of gifted students in our country are scarce, or old, and the differences in the results may be due to environmental factors [25], taking into account that the country in which subjects are educated can influence the results [44]. Aperribai and Garamendi [17] recommend working on these aspects in the school environment, especially as children grow up, and we have not found Spanish studies that evaluate self-esteem in gifted adolescents. Fourth, personal variables could help make wise choices and manage emotions. The main contribution of our work focuses on evaluating the perceived EI of students in addition to self-concept and self-esteem. EI could act as a protective factor for the psychological adjustment of students, favoring the appearance of prosocial behaviors and helping activate programs adapted to each group [30]. However, and fifth, we have not found studies that explore the possible differences in self-concept and self-esteem according to the different EI profiles among gifted students, whereas these studies do exist in regard to other groups with specific educational needs such as, for example, students with motor disabilities [113]. Delving into this type of relationships will contribute to theoretical development. Sixth, our research will help educators and health professionals identify potentially vulnerable children and adolescents. Having an understanding of the different profiles can be used to design more effective and specific interventions, aimed at improving the well-being of students in general and, of gifted students in particular, especially those at risk. Altogether, this will result in improving school coexistence and achieving more inclusive classrooms.

## 5. Limitations

Although representative, given the low percentage of identified students, a reduced sample was used. It is also a regional study, which has been carried out at a specific moment, with a specific population; it may not be possible to extrapolate the results. Moreover, it would be interesting to analyze the effect of culture and socioeconomic status in future studies or take into account the differences that identified students can present. The questionnaires used are self-reports. It would be interesting to contrast their self-evaluations with those of others who may be more objective and with ability tests. Within the academic self-concept dimension, the different subdimensions (mathematical, linguistic, etc.) have not been considered. It might be interesting to compare the results with an EI ability test. It would also be interesting to study gender differences, changes associated with age (especially distinguishing childhood from adolescence), etc. It would also be interesting to compare the results with other vulnerable groups.

## 6. Conclusions

For the development of talent, it is essential to consider the psychological and social aspects related to the teaching-learning processes. It is important to evaluate the self-concept and self-esteem of all students, and especially of those who are gifted, given that it seems that their potential often interferes with their well-being and can affect the development of their talent. To obtain a better academic self-concept, as in other studies, these students must improve their performance and to obtain higher grades they should understand their abilities better. For both issues, teacher involvement becomes vital.

Furthermore, having a good social self-concept and high self-esteem are key for professional performance, for cooperative work, and for exercising leadership tasks. The image that the subject has of their social performance and their own worth will condition their social networks and their participation in them and social support is essential for well-being. It therefore becomes necessary to better understand what risk and protective factors have an impact. Emotions play an important role in communication, the establishment of social contacts, and in one’s own social interaction with others. In addition, physical self-concept is equally important, especially during adolescence.

To guide the socio-emotional development of all students, socio-emotional learning processes are necessary, and adjusting interventions identifying vulnerable characteristics and profiles may be convenient. It would be interesting to offer programs that consider EI in more depth to strengthen the self-concept of these at-risk students, specifically those identified as gifted, curb the impact of possible attacks on self-esteem and self-concept, and provide them with effective coping strategies. Students must find the balance between feeling competent (intrapersonal) and getting along with peers (interpersonal), they must strive to be successful in tasks that present a challenge to them, but need strategies to be accepted by peers who do not understand them or who are upset by their results. To do this, teachers and educators must create environments that support the different strengths of students.

The most important source of social support is family; family cohesion is essential for life satisfaction, and therefore many of the educational actions and counseling should be aimed at improving their well-being when identified children are still young. Health services, psychologists and psychiatrists can also provide guidance and advice, including therapy if necessary.

## Figures and Tables

**Table 1:** Goodness of fit indices for confirmatory analysis of the three scales models.

	χ 2	g.l.	χ2/d.f.	NNFI	CFI	IFI	RMSEA
TMMS	393.35	249	1.58	0.90	0.90	0.90	0.04
RSE	71.61	27	2.65	0.90	0.92	0.92	0.08
AF5	241.14	160	1.50	0.91	0.92	0.92	0.04

Note: TMMS, Trait Meta-Mood Scale-24; RSE, Global Self-esteem Scale of Rosenberg; AF5, AF5 self-concept questionnaire; χ2, chi-square; df, degrees of freedom; IFI, Incremental Fit Index; NNFI, Non-Normed Fit Index; CFI, Comparative Fit Index; RMSEA, Root Mean Square Error of Approximation.

**Table 2:** Mean, standard deviation, and significate differences of the means of the total sample, gifted sample and unidentified students’ sample.

	Total Sample		Gifted Students		Unidentified Students			
	M	SD	M	SD	M	SD	*t*	ρ
Attention	2.97	0.83	3.67	0.78	3.01	0.80	0.84	0.40
Clarity	3.60	0.89	3.47	0.93	3.72	0.85	2.11	0.03
Repair	3.73	0.75	3.67	0.78	3.78	0.72	0.11	0.30
Self-esteem	4.10	0.69	4.00	0.72	4.20	0.65	2.13	0.03
Academic self-concept	4.28	0.78	4.27	0.84	4.30	0.71	0.26	0.80
Social self-concept	4.31	0.87	4.09	1.00	4.52	0.65	3.92	0.01
Emotional Self-concept	3.55	0.93	3.54	0.89	3.55	0.98	0.84	0.40
Family self-concept	4.61	0.63	4.50	0.70	4.71	0.52	2.63	0.01
Physical self-concept	3.96	0.73	3.82	0.75	4.10	0.70	2.90	0.01

Note: M = Mean; SD: Standard Deviation.

**Table 3:** Pearson correlations between dimensions of emotional intelligence.

	Gifted Students			Unidentified Students		
	Attention	Clarity	Repair	Attention	Clarity	Repair
Attention	1	0.21 *	0.27 **	1	0.22 *	0.26 **
Clarity	0.21 *	1	0.19 *	0.22 *	1	0.47 **
Repair	0.27 **	0.19 *	1	0.26 **	0.47 **	1

Note: * *p* < 0.05, two-tailed; ** *p* < 0.01, two-tailed.

**Table 4:** Values of the cluster centroids for the three-cluster solution.

	Gifted Students						Unidentified Students					
	Attention		Clarity		Repair		Attention		Clarity		Repair	
	M	SD	M	SD	M	SD	M	SD	M	SD	M	SD
Group 1	2.96	0.83	3.41	0.90	3.82	0.67	2.66	0.69	2.60	0.71	2.83	0.70
Group 2	2.21	0.59	4.33	0.69	2.44	0.48	3.26	0.65	4.00	0.63	4.1	0.48
Group 3	4.13	1.24	2.25	0.35	2.25	0.00	1.57	0.36	4.10	0.62	3.44	0.45
